# Interaction Between CYP1A2-Related Caffeine Metabolism and Vitamin B12/Folate Status in Patients with Metabolic Syndrome: A Novel Biomarker Axis

**DOI:** 10.3390/metabo15070450

**Published:** 2025-07-04

**Authors:** Laura Claudia Popa, Ahmed Abu-Awwad, Simona Sorina Farcas, Simona-Alina Abu-Awwad, Nicoleta Ioana Andreescu

**Affiliations:** 1 Doctoral School, “Victor Babes” University of Medicine and Pharmacy, Eftimie Murgu Square, No. 2, 300041 Timisoara, Romania; laura.popa@umft.ro; 2 Department of Microscopic Morphology, Discipline of Genetics, Genomic Medicine Centre “Victor Babes”, University of Medicine and Pharmacy, 300041 Timisoara, Romania; andreescu.nicoleta@umft.ro; 3 “Pius Brinzeu” Emergency Clinical County Hospital, Bld Liviu Rebreanu, No. 156, 300723 Timisoara, Romania; ahm.abuawwad@umft.ro; 4 Department XV—Discipline of Orthopedics—Traumatology, “Victor Babes” University of Medicine and Pharmacy, Eftimie Murgu Square, No. 2, 300041 Timisoara, Romania; 5 Research Center University Professor Doctor Teodor Sora, “Victor Babes” University of Medicine and Pharmacy, Eftimie Murgu Square, No. 2, 300041 Timisoara, Romania; 6 Department XII, Discipline of Obstetrics and Gynecology, “Victor Babes” University of Medicine and Pharmacy, Eftimie Murgu Square, No. 2, 300041 Timisoara, Romania; 7 “Louis Turcanu” Children Emergency Hospital, 300011 Timisoara, Romania

**Keywords:** metabolic syndrome, caffeine metabolism, cytochrome P-450 CYP1A2, vitamin B12, folic acid, personalized medicine

## Abstract

**Background/Objectives**: The prevalence of metabolic syndrome (MetS) is steadily increasing worldwide, driven by complex genetic, nutritional, and environmental factors. Caffeine metabolism, primarily mediated by CYP1A2 (though other enzymes such as CYP1A1 may also be involved), and the status of micronutrients such as vitamin B12 and folate have each been linked to MetS components. This study investigates the interaction between CYP1A2 genetic variants and vitamin B12/folate levels in patients with MetS, aiming to identify a novel biomarker axis with potential implications for personalized interventions. **Methods**: This cross-sectional observational study included 356 adults diagnosed with MetS, recruited from Western Romania. Genotyping for CYP1A2 rs762551 was performed using TaqMan PCR assays. Daily caffeine intake was assessed via validated dietary questionnaires. Serum levels of folate and vitamin B12 were measured using chemiluminescence immunoassays. **Results**: AA genotype patients with a moderate coffee intake (1–2 cups/day) had significantly higher folate and B12 levels than AC or CC carriers. These nutritional advantages were associated with more favorable BMI and triglyceride profiles. The interaction between CYP1A2 genotype and coffee intake was significant for both micronutrient levels and metabolic parameters, particularly in the AA group. No significant associations were found in high-coffee-intake subgroups (≥3 cups/day). **Conclusions**: The interplay between CYP1A2 polymorphisms and B-vitamin status may represent a clinically relevant biomarker axis in MetS. Moderate caffeine intake in slow metabolizers (AA genotype) may boost micronutrient status and metabolic health, supporting personalized nutrition.

## 1. Introduction

Metabolic syndrome (MetS) is a heterogeneous clinical entity defined by the coexistence of interconnected metabolic abnormalities, including central obesity, insulin resistance, dyslipidemia, and elevated blood pressure [1]. Rather than a singular disease, MetS reflects a systemic imbalance driven by complex interactions between genetic predisposition and environmental influences. This multifactorial nature complicates both early diagnosis and the development of targeted interventions, especially given the overlap of its components with other chronic disorders [2]. Although considerable progress has been made in identifying clinical markers, the molecular mechanisms underlying MetS remain incompletely understood. As a result, current research is shifting toward integrative approaches that explore novel biomolecular axes capable of linking metabolic dysfunction with modifiable lifestyle and dietary factors [3,4].

Among the metabolic pathways currently under investigation, the metabolism of caffeine, ubiquitously consumed worldwide, has drawn particular attention due to its interaction with cytochrome P450 enzymes, especially CYP1A1 and CYP1A2. These hepatic enzymes are responsible for the primary demethylation of caffeine, but they also participate in various oxidative and detoxification processes that may influence systemic metabolic regulation. Variability in CYP1A1 and CYP1A2 activity, influenced by both genetic polymorphisms and environmental exposures (such as smoking, diet, or xenobiotics), has been shown to affect not only caffeine clearance but also the generation of reactive oxygen species and pro-inflammatory mediators [5,6].

Concurrently, micronutrients such as vitamin B12 and folate play essential roles in one-carbon metabolism, DNA synthesis, and homocysteine regulation [7,8]. Deficiencies in either vitamin have been associated with metabolic and vascular dysfunctions, including increased oxidative stress, endothelial impairment, and insulin resistance, all features commonly seen in patients with MetS [7]. Importantly, the interdependence between methylation status and hepatic enzyme function remains a relatively unexplored area in metabolic research [9].

While both caffeine metabolism and B-vitamin status have individually been implicated in the pathophysiology of MetS, the potential interaction between these two systems has received limited attention. The possibility that CYP1A1 and CYP1A2 enzyme activity could impact the effects of vitamin B12 and folate provides a new avenue for research, offering valuable insights into the mechanisms of metabolic regulation in affected patients. Furthermore, considering the accessibility and modifiability of both caffeine intake and micronutrient levels, exploring their relationship may hold practical value for early detection or risk stratification.

The aim of this study is to investigate the interaction between CYP1A2-related caffeine metabolism and vitamin B12/folate status in individuals diagnosed with metabolic syndrome. By analyzing correlations between enzyme activity, micronutrient levels, and key metabolic parameters, we seek to identify whether this biochemical interplay constitutes a relevant biomarker axis that could inform both clinical assessment and personalized intervention strategies in metabolic syndrome management.

## 2. Materials and Methods

### 2.1. Study Design and Population

This was a cross-sectional observational study. Patients included in this study were recruited from the western region of Romania, primarily from Timis County, and were evaluated at a tertiary medical center. Genetic testing was performed at the Department of Genetics at the ‘Victor Babeș’ University of Medicine and Pharmacy, Timișoara.

Biochemical analyses were conducted at a private, accredited laboratory.

A total of 356 patients were included in the final analysis, all of whom had available clinical, biochemical, and genetic data relevant to the study objectives. Using established criteria from the International Diabetes Federation (IDF) [10] and the National Cholesterol Education Program Adult Treatment Panel III (NCEP ATP III) [11], we identified 320 patients (89.9%) who met the diagnostic threshold for metabolic syndrome, defined by the presence of at least three of the following five components: obesity (BMI ≥ 30), hypertriglyceridemia (≥150 mg/dL), low HDL cholesterol (sex-specific thresholds), elevated fasting glucose (≥100 mg/dL or known diabetes), and hypertension [12].

This study was conducted in accordance with the Declaration of Helsinki and was approved by the Ethics Committee for Scientific Research of the ‘Victor Babeș’ University of Medicine and Pharmacy, Timișoara (approval no. 86/2020). All participants provided written informed consent prior to their inclusion in the study. Ethical considerations included voluntary participation, confidentiality of personal data, and clear communication of potential risks and benefits associated with the study procedures. Participants had the right to withdraw at any point without consequences, and data were handled and stored securely to protect participants’ identities and confidentiality.

The inclusion criteria were as follows:

- Adult patients aged between 18 and 75 years.
- Confirmed diagnosis of metabolic syndrome according to International Diabetes Federation (IDF) and National Cholesterol Education Program Adult Treatment Panel III (NCEP ATP III) criteria, requiring at least three of the following five components [11,12,13]:
  ○ Central obesity (BMI ≥ 30 kg/m^2^).
  ○ Elevated serum triglycerides (≥150 mg/dL).
  ○ Low HDL cholesterol levels (<40 mg/dL in men and <50 mg/dL in women).
  ○ Elevated fasting blood glucose (≥100 mg/dL or previously diagnosed type 2 diabetes).
  ○ Elevated blood pressure (≥130/85 mmHg) or current antihypertensive treatment.
- Availability of complete clinical and biochemical data necessary for the study.
- Availability of genetic data, specifically genotyping results for the CYP1A2 gene SNP rs762551.
- Adequate completion of questionnaires regarding daily caffeine consumption.
- Provision of signed written informed consent for voluntary participation in the study.

The exclusion criteria were as follows:

- Age under 18 or over 75 years.
- Pregnant or breastfeeding women.
- Confirmed diagnosis of severe hepatic impairment or liver cirrhosis.
- Advanced chronic kidney disease (eGFR < 30 mL/min/1.73 m^2^) [13].
- Severe acute or chronic inflammatory diseases, including active autoimmune diseases or chronic infections (e.g., HIV, Hepatitis B or C).
- Recent history (within the past 6 months) of major surgical interventions or prolonged hospitalizations (>10 days).
- Chronic pharmacological treatment known to significantly interfere with caffeine metabolism or vitamin status (e.g., anticonvulsants, antituberculosis medication, recent chemotherapy).
- Severe digestive disorders (Crohn’s disease, ulcerative colitis, gastric bypass) affecting vitamin B12 and folate absorption.
- Excessive alcohol intake (>2 units/day for women and >3 units/day for men) or history of substance abuse within the past year.

### 2.2. Data Collection and Clinical Variables

Sociodemographic, anthropometric, and clinical data were collected from structured clinical records and interviews. Weight and height were measured using calibrated instruments, and body mass index (BMI) was calculated accordingly. Blood pressure measurements were obtained using automated oscillometric devices. Medication history, including statins, fibrates, antihypertensives, antidiabetic drugs, anticoagulants, and antiplatelet therapy, was documented.

To ensure compliance with the General Data Protection Regulation (GDPR), all personal data collected from participants were coded and anonymized at the initial stage of data handling. Each participant was assigned a unique study identifier, which replaced any personal identifying information in the dataset. The master key linking identifiers to personal details was stored securely and separately from the research dataset, accessible only to the principal investigator and authorized personnel.

All electronic data were stored on secured servers with controlled access, requiring password authentication and two-factor authentication measures. Physical data (such as informed consent forms and questionnaire records) were secured in locked filing cabinets within a restricted-access office. Regular audits were conducted to ensure compliance with data protection protocols and to verify the integrity and confidentiality of participant data throughout the study period.

Access to research data was strictly limited to research team members trained in GDPR compliance, data protection protocols, and confidentiality requirements. Additionally, any publication or dissemination of study results was carried out exclusively using aggregated or anonymized data to safeguard participants’ identities. All data handling and storage procedures adhered to institutional guidelines approved by the Ethics Committee of the ‘Victor Babeș’ University of Medicine and Pharmacy, Timișoara.

### 2.3. Biochemical Assessment

Fasting blood samples were collected in the morning after at least 10 h of fasting. Serum concentrations of glucose, cholesterol, triglycerides, creatinine, and potassium were determined using standard automated laboratory methods [14]. Serum folate and vitamin B12 levels were measured using chemiluminescent immunoassays [15].

Blood samples were collected from participants after a minimum fasting period of 10 h. Samples were drawn into standardized blood collection tubes: EDTA tubes (BD Vacutainer^®^, lavender top, Becton, Dickinson and Company, Franklin Lakes, NJ, USA) for plasma and whole blood and serum separator tubes (BD Vacutainer^®^, gold top) for serum [16]. Immediately after collection, serum samples were allowed to clot at room temperature for 30 min before centrifugation.

All samples were centrifuged at 3500 rpm for 15 min at room temperature (approximately 20–22 °C) to separate plasma and serum from cellular components. Subsequently, aliquots of serum and plasma were carefully transferred to labeled cryovials, avoiding hemolysis. The aliquoted samples were promptly stored at −80 °C until further analysis to maintain the integrity and stability of biological markers [17].

### 2.4. Assessment of Daily Caffeine Consumption

The daily caffeine intake of participants was assessed through structured questionnaires. Participants provided detailed information about their daily coffee consumption, specifying the amount consumed (in milliliters), whether the coffee was caffeinated or decaffeinated, and any additional ingredients such as milk. To accurately quantify caffeine consumption, a 24 h dietary recall method was employed over four non-consecutive days, including three weekdays and one weekend day, to capture habitual intake and account for potential variations throughout the week [18]. During these recalls, trained medical personnel or research assistants conducted structured interviews to document all food and beverage intake. Subsequently, reported intakes were converted into caffeine values using the Nutrio web application (Naturalpixel SRL, Bucharest, Romania, https://nutritioapp.com, accessed on 27 March 2020), enabling a precise calculation of daily caffeine consumption based on standardized nutrient databases.

### 2.5. SNP Selection and Genotyping

Genomic DNA was isolated from whole-blood samples using the MagCore Nucleic Acid Extraction Kit provided by RBC Bioscience (New Taipei City, Taiwan), following the manufacturer’s standard guidelines [19]. The concentration of the extracted DNA was then measured using an Epoch Microplate Spectrophotometer from Agilent BioTek (Santa Clara, CA, USA).

In our investigation, we focused on the CYP1A2 single-nucleotide polymorphism (SNP) rs762551, chosen for its well-established role in caffeine metabolism. Genotyping of this specific SNP was carried out by using the TaqMan Real-Time PCR assay (assay ID C_8881221) and Taqman Genotyping Master Mix (TaqMan™ Genotyping Master Mix (cat. no. 4371355, Thermo Fisher Scientific, Life Technologies, Carlsbad, CA, USA) from Life Technologies, Applied Biosystems (Thermo Fisher Scientific, Waltham, MA, USA). Complete details of the protocol were carefully followed according to the guidelines specified in the Applied Biosystems TaqMan SNP Genotyping Assays manual [20].

The purified DNA samples underwent amplification reactions on a LightCycler 480 Real-Time PCR System (Roche Diagnostics), employing the Gene Scanning software, version 1.5.1. All reactions were uniformly conducted in 96-well plates, each with a consistent volume of 20 µL to standardize DNA concentrations across samples.

The employed TaqMan assay involved two specific primers designed to amplify the target genetic sequence, each labeled with distinct fluorescent dyes to differentiate between the genetic variants. Upon the completion of PCR cycles, endpoint fluorescence readings were captured directly by the real-time PCR equipment.

To ensure accuracy and eliminate bias, genotyping was independently executed by two researchers who remained blinded to sample identities. Additionally, 5% of the DNA samples were randomly selected for further validation.

### 2.6. Statistical Analysis

Statistical analyses were performed using GraphPad Prism version 6 (GraphPad Software, San Diego, CA, USA). Descriptive statistics were employed to summarize demographic and clinical characteristics, presented as means ± standard deviations (SDs) or proportions (%), as appropriate. Comparisons among genotype groups (AA, AC, CC) and coffee consumption levels (moderate versus high) were made using one-way ANOVA for continuous variables, followed by Tukey’s multiple-comparison test to identify specific group differences.

Correlations between nutritional biomarkers (folate, vitamin B12) and coffee consumption stratified by genotype were evaluated using Pearson’s correlation coefficient, with statistical significance determined by the associated *p*-values. Multivariate regression analysis was conducted to investigate the independent and interactive effects of genotype (AA versus AC + CC), coffee consumption levels, and micronutrient status (folate and vitamin B12) on metabolic parameters. Beta coefficients (β), standard errors, and *p*-values were reported, with statistical significance established at a threshold of *p* < 0.05.

The normality of data distribution was assessed using the Shapiro–Wilk test prior to applying parametric statistical tests. Variables that did not meet the assumption of normality were either log-transformed or analyzed using appropriate non-parametric methods, as applicable.

Figures illustrating the relationships between serum folate and vitamin B12 levels and body mass index (BMI), stratified by genotype and coffee intake, were generated using scatter plots. All tests performed were two-tailed, and significance was consistently set at a level of *p* < 0.05.

## 3. Results

The demographic and lifestyle characteristics of study participants were analyzed according to their CYP1A2 genotype categories (AA, AC, CC) (Table 1).

The variables examined included age, BMI, anthropometric parameters, gender distribution, smoking habits, sedentary behavior, and the prevalence of family history of cardiovascular disease (CVD).

Observed differences across genotype groups suggest possible genotype-specific influences on select demographic factors and familial predisposition, while lifestyle behaviors showed comparable patterns among the groups.

Table 2 presents a comparative analysis of clinical and demographic variables between individuals with the AA genotype and those with combined AC and CC genotypes (AC + CC). A statistically marginal difference was observed in age (*p* = 0.046). Other variables such as BMI, weight, height, and categorical factors (sex, smoking, sedentary lifestyle, and family history of CVD) showed no statistically significant differences, indicating a comparable distribution across these groups.

The analysis in Table 3 explores the association between levels of coffee consumption (moderate vs. high intake) and key nutritional and metabolic biomarkers among the study participants. Individuals were grouped according to their reported daily coffee intake (1–2 cups/day versus ≥ 3 cups/day), and serum biomarkers, including folate and vitamin B12 concentrations, were assessed alongside anthropometric and metabolic parameters such as BMI, glucose, triglyceride, and HDL cholesterol levels. Significant differences in folate (*p* = 0.004) and vitamin B12 (*p* = 0.012) between the coffee consumption groups suggest a potential dose-dependent relationship. In contrast, metabolic variables related to glucose and lipid profiles demonstrated no substantial variation based on coffee intake.

Table 4 presents comparative data on folate, vitamin B12, and triglyceride levels stratified by coffee consumption (moderate versus high) and CYP1A2 genotype groups (AA versus AC + CC). Within the moderate coffee consumption category, statistically significant differences between genotype groups were observed for folate (*p* = 0.023) and vitamin B12 levels (*p* = 0.041), suggesting that genetic variations may influence nutrient metabolism at lower coffee intake levels. Conversely, among participants with higher coffee consumption, differences between genotypes in folate (*p* = 0.178), vitamin B12 (*p* = 0.215), and triglyceride levels (*p* = 0.092) were not statistically significant, indicating that the impact of genotype variations on these biomarkers may diminish at increased levels of coffee consumption.

The correlations between coffee consumption and serum levels of folate and vitamin B12, differentiated by CYP1A2 genotype groups (AA vs. AC + CC) at moderate versus high coffee intake levels, are presented in Table 5. Positive and statistically significant correlations emerged exclusively among individuals with moderate coffee consumption carrying the AA genotype (folate: r = 0.284, *p* = 0.015; vitamin B12: r = 0.253, *p* = 0.032), highlighting a potential genotype-dependent beneficial impact of moderate coffee intake on nutritional biomarkers. In contrast, among individuals with high coffee intake, correlations were weak and not statistically significant (e.g., for AA genotype: folate *p* = 0.243; vitamin B12 *p* = 0.332), suggesting that the influence of genotype on the relationship between coffee consumption and nutrient levels diminishes as coffee intake increases.

Multivariate regression analysis revealed significant main effects of CYP1A2 genotype (*p* = 0.032), coffee consumption level (*p* = 0.027), folate (*p* = 0.001), and vitamin B12 (*p* = 0.015) on metabolic parameters (Table 6). Significant two-way interactions were also observed for genotype × coffee consumption (*p* = 0.049), genotype × folate (*p* = 0.025), and coffee consumption × folate (*p* = 0.012), indicating a complex interplay between genetic and nutritional factors. Three-way interactions showed non-significant trends (*p* > 0.05), suggesting subtle effects that require further investigation.

**Figure 1:**
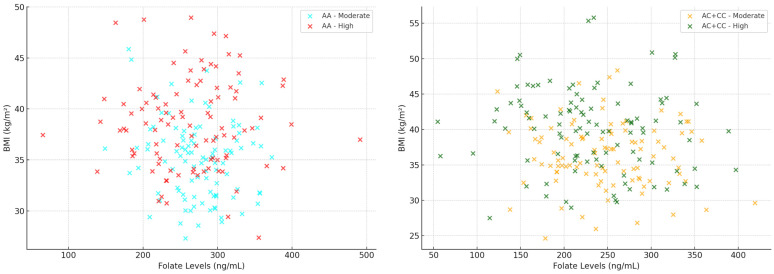
Relationship between serum folate levels and BMI stratified by CYP1A2 genotype (AA vs. AC + CC) and coffee consumption (moderate vs. high).

The scatter plots shown in Figure 1 illustrate the relationship between serum folate levels and body mass index (BMI), stratified by CYP1A2 genotype (AA vs. AC + CC) and daily coffee consumption (moderate vs. high). In the AA genotype group (left plot), individuals with moderate coffee intake generally display higher folate concentrations and relatively lower BMI compared to those with high coffee consumption. Conversely, the AC + CC genotype group (right plot) shows lower serum folate levels associated with higher BMI, particularly among individuals with high coffee intake. Regarding correlation patterns, a more noticeable beneficial association between folate levels and BMI is evident in participants with the AA genotype and moderate coffee consumption. In contrast, these associations become weaker or non-existent among individuals with higher coffee intake or those carrying the AC + CC genotype. This trend reinforces the hypothesis of a genotype-dependent interplay between dietary habits and nutritional status, underscoring the potential importance of tailored dietary recommendations based on genetic profiles to effectively manage metabolic syndrome.

**Figure 2:**
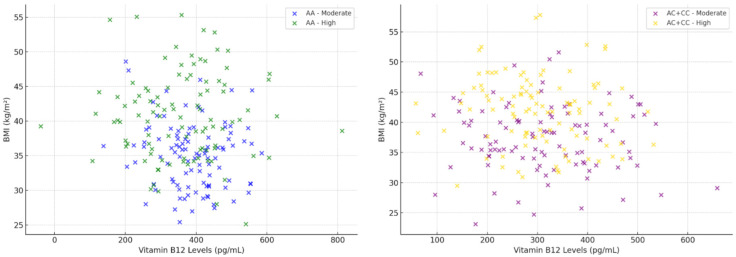
Relationship between serum vitamin B12 levels and BMI stratified by CYP1A2 genotype (AA vs. AC + CC) and coffee consumption (moderate vs. high).

The two scatter plots in Figure 2 illustrate the relationship between serum vitamin B12 levels and body mass index (BMI), stratified by CYP1A2 genotype (AA vs. AC + CC) and daily coffee consumption (moderate vs. high). In the AA genotype group (left plot), individuals with moderate coffee intake tend to have higher vitamin B12 levels and relatively lower BMI compared to those with high coffee consumption. Conversely, participants with the AC + CC genotype (right plot) generally exhibit lower vitamin B12 levels associated with higher BMI, regardless of coffee consumption level. Regarding correlations, a clearer positive relationship is observed among participants with the AA genotype and moderate coffee consumption, suggesting a beneficial interaction between moderate caffeine intake and genetic caffeine metabolism capacity on vitamin B12 status and BMI. This correlation is considerably weaker and non-significant among individuals with high coffee intake and those with the AC + CC genotype. These observations support the hypothesis of a complex, genotype-specific interaction between dietary intake and nutritional status, emphasizing the importance of tailoring dietary and therapeutic interventions to individual genetic profiles.

## 4. Discussion

The results of our study bring a fresh perspective on the complex interactions between caffeine metabolism, vitamin B12 and folate status, and the clinical features of MetS, highlighting the role of genetic polymorphisms in the CYP1A1 and CYP1A2 genes. The study shows that individuals with the AA gen0otype of CYP1A2 who drink coffee moderately have significantly higher levels of B12 and folate, which are associated with a more favorable metabolic profile [21,22,23].

A number of studies in the scientific literature have pointed out the importance of cytochrome P450 enzymes—especially the CYP1A1 and CYP1A2 isoforms—in the hepatic metabolism of caffeine [24]. CYP1A2 is considered the main enzyme responsible for breaking down caffeine in the liver, handling over 90% of its demethylation reactions, while CYP1A1 plays a smaller but still meaningful role under certain physiological or pathological conditions. The activity of these enzymes is affected by several factors, including exposure to exogenous inducers (like smoking or eating cruciferous vegetables), as well as genetic variability, which leads to a wide range of individual metabolic rates [5,25].

One of the best-known genetic polymorphisms that affects CYP1A2 activity is the SNP rs762551 (also known as -163C > A). This polymorphism directly impacts gene expression and, as a result, enzyme activity [26,27]. Several studies have shown that individuals with the AA genotype (homozygous for the A allele) have lower enzyme activity, meaning they metabolize caffeine more slowly. In contrast, people with the AC or CC genotypes tend to have higher CYP1A2 activity and thus metabolize caffeine more quickly [24,26].

This kind of genetic variability has important implications for how caffeine affects the body, influencing individual responses to its consumption—ranging from how long the stimulating effect lasts to the potential cardiovascular risks or effects on sleep [28,29].

For example, epidemiological studies suggest that slow metabolizers (AA genotype) may be more vulnerable to the adverse effects of caffeine, like increased blood pressure, while fast metabolizers (CC genotype) might actually benefit from the protective effects of moderate coffee intake [30].

Our study results are in line with these findings from the literature, supporting the idea that the AA genotype of the rs762551 SNP is linked to slower caffeine metabolism [31]. This has important nutritional and clinical implications, pointing toward the need for a more personalized approach when it comes to caffeine intake recommendations, based on a person’s genetic profile.

From a physiological point of view, the mechanisms through which genetic polymorphisms—especially the rs762551 variant of the CYP1A2 gene—influence individual metabolic profiles are complex and multifactorial. These mechanisms involve not only the direct metabolism of caffeine but also indirect effects through interactions with the metabolism of B-complex vitamins, particularly vitamin B12 and folic acid (folate), which are important for homocysteine metabolism and DNA methylation. Caffeine, through its effects on liver enzymes, may indirectly impact the availability and use of these essential vitamins, thereby influencing key metabolic pathways that help maintain metabolic homeostasis [32,33,34].

Vitamin B12 and folate play a major role in the methylation cycle, acting as essential cofactors in the conversion of homocysteine into methionine—a key step for the production of S-adenosylmethionine (SAM), the body’s main methyl group donor. This process is crucial for gene expression regulation through DNA methylation, DNA repair, and neurotransmitter synthesis. A deficiency in B12 or folate leads to an accumulation of homocysteine in the blood (hyperhomocysteinemia), which is known as an independent risk factor for cardiovascular disease, endothelial dysfunction, and potentially contributes to insulin resistance and the development of metabolic syndrome [7,35,36].

In this context, the rs762551 polymorphism may indirectly affect the levels of these vitamins by influencing the rate of caffeine metabolism. Specifically, the AA genotype, associated with a slow metabolizer phenotype, might lead to lower hepatic enzyme induction and thus slower elimination of co-metabolized or interacting nutrients. This idea is supported by studies showing that slow caffeine metabolism is linked to reduced excretion of water-soluble compounds, including B-complex vitamins. So, slower metabolism could be tied to a higher retention of vitamin B12 and folate, which may positively affect homocysteine balance and support potentially beneficial epigenetic processes [7,24,27].

The data from our study support this perspective, suggesting that individuals with the AA genotype tend to have a more favorable metabolic profile when it comes to the availability of vitamin B12 and folate. This might help explain some of the significant differences seen in metabolic markers related to MetS, compared to individuals with the AC or CC genotypes. These findings could have important implications for personalized nutrition strategies, pointing to the idea that people who metabolize caffeine slowly might actually benefit from a moderate caffeine intake—assuming they maintain a good vitamin status.

Our observations about the link between moderate coffee consumption, the AA genotype, and better levels of B12 and folate are also supported by other recent studies. One study emphasizes the importance of including genetic testing in research on caffeine and its health effects, while another reports similar associations between moderate caffeine intake and protective effects on certain metabolic parameters depending on CYP1A2 gene variations [37].

The correlation and multivariate regression analysis in our study showed that the interaction between caffeine intake and CYP1A2 genotypes significantly influences serum vitamin levels and metabolic markers. Especially, the significant associations found only in people with the AA genotype and moderate caffeine consumption highlight the complexity of gene–nutrition interactions. They suggest that the beneficial effects of B vitamins may be more noticeable in the context of slow caffeine metabolism [38].

From a clinical perspective, these findings offer a valuable opportunity to personalize nutritional and therapeutic recommendations based on an individual’s genetic profile. Patients with the AA genotype might benefit from moderate coffee consumption as part of a balanced diet, considering the observed association with higher levels of folate and vitamin B12—which could, in turn, improve their overall metabolic profile. On the other hand, patients with AC or CC genotypes, who metabolize caffeine more quickly, may require tailored nutritional strategies to maintain optimal levels of these vitamins and avoid possible negative effects of high caffeine intake on vitamin status.

Among the strengths of this study are a relevant sample size, the use of validated and accurate tools for both nutritional and genetic assessment, and a robust, comprehensive statistical approach. The applied methodology also allowed us to identify and better understand the complex interactions between genetics, nutrition, and metabolic parameters, adding valuable insights to the current scientific literature.

However, there are also some notable limitations. The cross-sectional design does not allow for conclusions about causality—it only shows associations between variables. Nutritional data were based on self-reported intake, which can introduce subjectivity and errors in estimating actual consumption. Additionally, the lack of evaluation of other dietary factors or lifestyle elements may influence how the results should be interpreted.

Given the high prevalence of metabolic syndrome and the widespread consumption of caffeine, these findings could contribute to the development of more personalized public health recommendations—especially concerning caffeine intake and micronutrient needs based on genetic background.

Looking ahead, future research should include longitudinal and interventional studies to clearly establish causal links between the variables studied. It would also be useful to assess more detailed molecular markers of caffeine and vitamin metabolism, including gene expression and specific enzymatic activity, to better understand the physiological mechanisms involved. Further studies should expand genetic analysis by including other relevant variants and exploring their interactions with different dietary factors and lifestyle habits.

## 5. Conclusions

Our study showed that patients with metabolic syndrome who have the AA genotype for the rs762551 polymorphism in the CYP1A2 gene and drink coffee in moderate amounts (1–2 cups/day) have significantly higher levels of vitamin B12 and folate compared to those with AC or CC genotypes. These differences were noticeable only with moderate coffee intake, not with high intake (≥3 cups/day), which suggests a dose- and genotype-dependent effect. Correlation and multivariate regression analyses confirmed that the interaction between genotype and caffeine intake significantly influences the serum levels of these vitamins, with possibly beneficial effects on metabolic markers, especially triglyceride levels and body mass index. These findings support the idea of a synergistic effect between slow caffeine metabolism and maintaining an optimal vitamin status in the context of metabolic syndrome.

## Figures and Tables

**Table 1:** Baseline demographic and clinical characteristics according to CYP1A2 genotype distribution.

Variable	AA (*n* = 66)	AC (*n* = 145)	CC (*n* = 109)	*p* Value	*p* Value (AA–AC)	*p* Value (AA–CC)	*p* Value (AC–CC)
Age (years) *	54.9 ± 7.8	55.0 ± 5.9	53.4 ± 8.1	0.121	0.103	0.942	0.085
BMI *	37.0 ± 3.2	36.6 ± 2.9	37.2 ± 3.2	0.103	0.149	0.724	0.051
Weight (kilograms) *	104.9 ± 16.3	102.2 ± 15.9	105.5 ± 16.1	0.465	0.223	0.415	0.639
Height (meters) *	1.68 ± 0.09	1.67 ± 0.09	1.68 ± 0.1	0.929	0.711	0.952	0.772
Sex (F) **	33 (50.00%)	77 (53.10%)	56 (51.37%)	0.909	0.678	0.786	0.861
Smokers **	20 (30.30%)	57 (39.31%)	38 (34.86%)	0.434	0.201	0.534	0.469
Sedentary lifestyle **	36 (54.54%)	68 (46.89%)	52 (47.70%)	0.570	0.306	0.384	0.899
Family history of CVD ^(a)^ **	34 (51.51%)	97 (66.89%)	67 (61.46)	0.103	0.034	0.202	0.375

* Mean ± SD; ** percentage; ^(a)^ CVD = cardiovascular disease.

**Table 2:** Comparison of clinical and demographic variables between AA and AC + CC genotype groups.

Variable	AA (*n* = 66)	AC + CC (*n* = 254)	*p* Value
Age (years) *	54.9 ± 7.8	54.31 ± 6.93	0.046
BMI *	37.0 ± 3.2	36.86 ± 3.03	0.301
Weight (kilograms) *	104.9 ± 16.3	103.62 ± 15.99	0.432
Height (meters) *	1.68 ± 0.09	1.674 ± 0.09	0.670
Sex (F) **	33 (50.00%)	133 (52.36)	0.735
Smokers **	20 (30.30%)	95 (37.40%)	0.274
Sedentary lifestyle **	36 (54.54%)	120 (47.24)	0.294
Family history of CVD ^(a)^ **	34 (51.51%)	164 (64.56%)	0.061

* Mean ± SD; ** percentage; ^(a)^ CVD = cardiovascular disease.

**Table 3:** Metabolic and nutritional parameters stratified by daily coffee consumption.

Parameter	1–2 Cups/Day (*n* = 180)	≥3 Cups/Day (*n* = 140)	*p* Value
Folate (ng/mL)	285.1 ± 145.6	259.0 ± 132.1	0.004
Vitamin B12 (pg/mL)	4.1 ± 3.2	3.5 ± 2.9	0.012
BMI (kg/m^2^)	36.9 ± 6.5	37.4 ± 6.8	0.485
Glucose (mg/dL)	105.2 ± 22.4	107.5 ± 24.0	0.362
Triglycerides (mg/dL)	160.3 ± 58.9	165.7 ± 61.2	0.392
HDL Cholesterol (mg/dL)	46.7 ± 11.2	45.9 ± 10.8	0.491

**Table 4:** Interaction between coffee consumption and CYP1A2 genotypes on nutritional and lipid profiles.

Coffee Consumption	Genotype	Folate (ng/mL)	Vitamin B12 (pg/mL)	Triglycerides (mg/dL)
Moderate (1–2 cups/day)	AA	300.2 ± 145.0	4.3 ± 3.0	155.2 ± 59.0
Moderate (1–2 cups/day)	AC + CC	271.0 ± 140.3	3.8 ± 2.8	162.0 ± 57.1
*p* Value		0.023	0.041	0.365
High (≥3 cups/day)	AA	260.1 ± 130.4	3.6 ± 2.9	158.7 ± 60.3
High (≥3 cups/day)	AC + CC	245.7 ± 125.9	3.2 ± 2.5	170.4 ± 62.2
*p* Value		0.178	0.215	0.092

AA—slow metabolizers; AC + CC—intermediate/rapid metabolizers.

**Table 5:** Correlation between nutritional biomarkers and coffee consumption stratified by CYP1A2 genotype.

Coffee Consumption	Combined CYP1A2 Genotype	Folate Correlation (r)	*p*-Value Folate	B12 Correlation (r)	*p*-Value B12
Moderate (1–2 cups/day)	AA	0.284	0.015	0.253	0.032
Moderate (1–2 cups/day)	AC + CC	0.149	0.130	0.107	0.291
High (≥3 cups/day)	AA	0.123	0.243	0.098	0.332
High (≥3 cups/day)	AC + CC	0.083	0.409	0.051	0.604

**Table 6:** Multivariate regression analysis of CYP1A2 genotype, coffee consumption, and vitamin status on metabolic parameters in metabolic syndrome.

Predictor	β Coefficient	Std. Error	*p*-Value	Significance
Main effects				
Genotype (AA vs. AC + CC)	0.15	0.07	0.032	Yes
Coffee Consumption (Moderate vs. High)	−0.18	0.08	0.027	Yes
Folate (ng/mL)	−0.035	0.01	0.001	Yes
Vitamin B12 (pg/mL)	−0.022	0.009	0.015	Yes
Interactions (2-way)				
Genotype × Coffee Consumption	0.125	0.065	0.049	Yes
Genotype × Folate	−0.018	0.008	0.025	Yes
Genotype × Vitamin B12	−0.012	0.007	0.097	No
Coffee Consumption × Folate	−0.024	0.009	0.012	Yes
Coffee Consumption × Vitamin B12	−0.015	0.008	0.068	No
Interactions (3-way)				
Genotype × Coffee Consumption × Folate	0.011	0.006	0.081	No
Genotype × Coffee Consumption × Vitamin B12	0.008	0.005	0.120	No

## Data Availability

Data Availability Statements are available upon request from the corresponding author.
